# Determine the myocardial T2* cut-off value in thalassemia using gaussian mixtures models

**DOI:** 10.1186/1532-429X-14-S1-O77

**Published:** 2012-02-01

**Authors:** Taigang He, Rong R Liu, Yanqiu Feng, John-Paul Carpenter, Yongrong Lai, Dudley J Pennell, David N Firmin

**Affiliations:** 1 Biomedical Research Unit, Royal Brompton Hospital, London, UK; 2 School of Biomedical Engineering, Southern Medical University, Guangzhou, China; 3 Department of Hematology, The First Affiliated Hospital of Guangxi Medical University, Nanning, China

## Background

Cardiovascular magnetic resonance (CMR) can provide a non-invasive means of measuring the amount of tissue iron. The iron results in shortening of proton relaxation times and both CMR T2* and T2 have been validated as non-invasive means for assessment of myocardial iron overload. (1,2,3,4). We have reported that a T2* value below 20ms represents iron overload in the heart (5). This cut-off value, though proven very useful in clinical practice, was based on 15 normal volunteers in our initial report (5). There is currently little data to confirm this important cut-off value which has been widely accepted for early diagnosis of myocardial iron in patients with thalassemia major (TM).

A mixture model corresponds to the mixture distribution that represents the probability distribution of observations in the overall population. We believe myocardial T2* and T2 data may contain an intrinsic pattern which can be explored for clinical diagnosis. The current study, therefore, aimed at clustering patients automatically into normal and abnormal groups by using Gaussian mixture models (6).

## Methods

In total 236 TM patients (age 28±20 years old, 119 males) were studied on a 1.5T MRI scanner All patients were scanned using the black blood T2* (3) and T2 (4) sequences, each within a breath-hold. A single mid-ventricular short axis slice was imaged with T2* and T2 measured in the left ventricular septum. The clustering algorithm using Gaussian mixture models was developed in Matlab.

## Results

**Figure 1:**
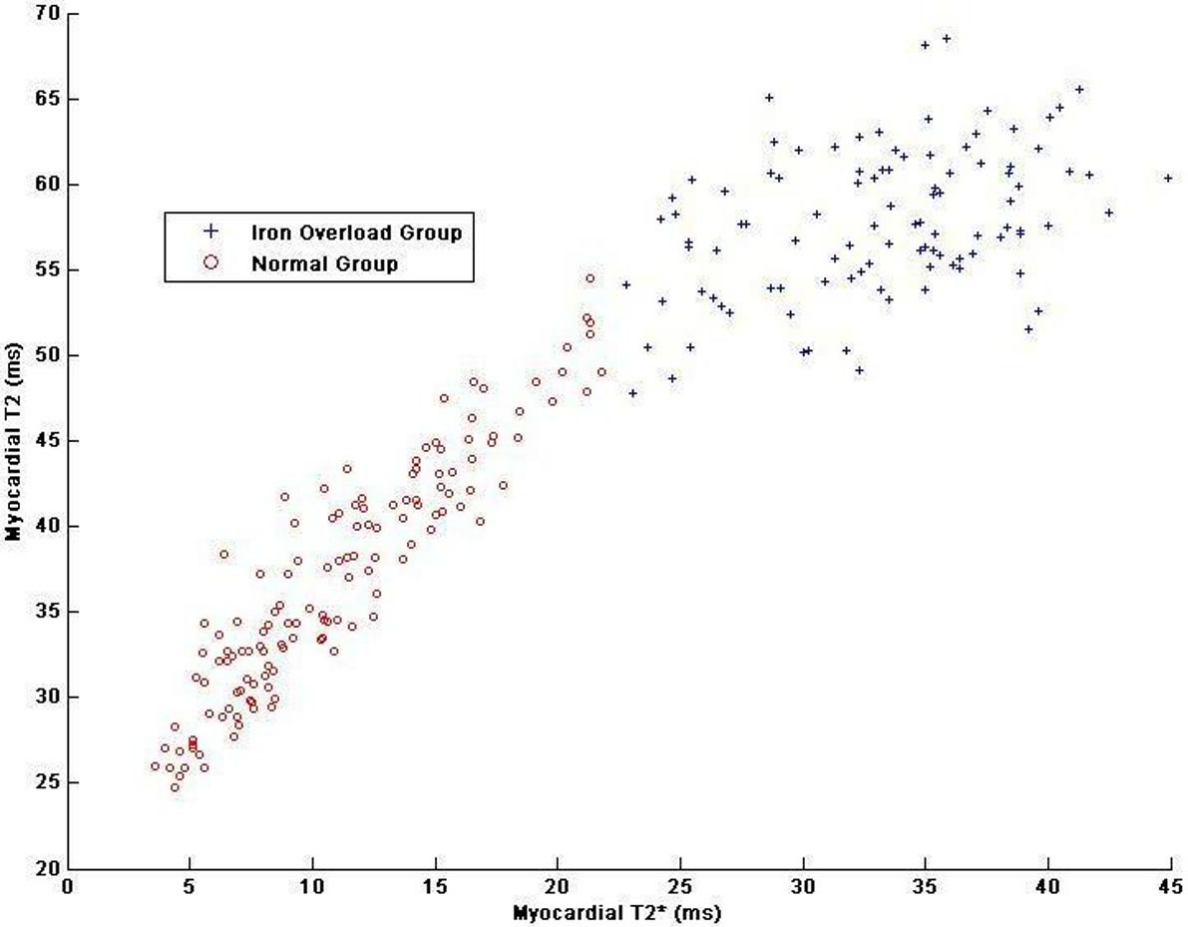
Automated clustering of patients with thalassemia major (n=236). T2* value of 20ms is a good indicator of patients with myocardial iron overload. There are mixtures between 20ms<T2*<25ms but the number is very limited.

**Figure 2:**
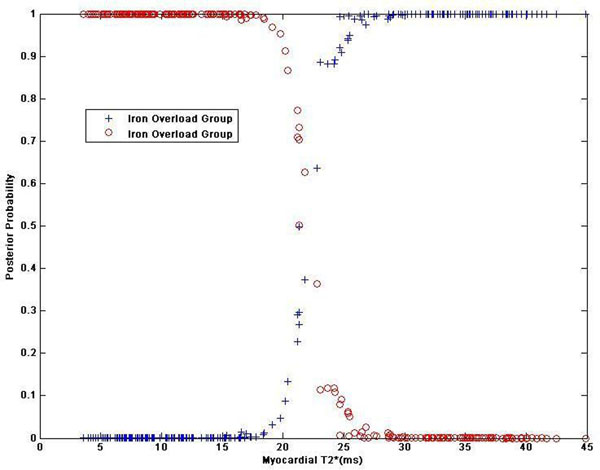
The posterior probabilities for soft clustering. Membership scores, set as the posterior probabilities, describe how similar each point is to each cluster's archetype. The result demonstrate that the fitted distribution separates the data into two groups nicely. Very few points have scores close to 0.5.

Figure 1 shows the automated classification of T2* and T2 data into two groups (red and blue) using Gaussian mixture models. This automated clustering result agrees well with that of using T2* cut-off value of 20ms. Figure 2 shows the posterior probability of each data point, which confirm Gaussian mixed mmodels can well represent distributions of myocardial T2* and T2 measurements in thalassemia.

## Conclusions

This study confirms that T2* is a reliable tool for screening patients with iron overload. There appears an intrinsic pattern in myocardial T2* and T2 data in patients with thalassemia patients; Gaussian mixture model can automatically cluster the 2-dimensional data and the results agree well with that using a T2* cut-off value of 20ms. There are a few mismatched samples and a further study is needed to clarify and explain this phenomimen in order to provide improved myocardial tissue characterization for patients with TM.

## Funding

This study was supported by the UK NIHR Cardiovascular Biomedical Research Unit of Royal Brompton Hospital and Imperial College, London. Dr He was supported by Wellcome Trust Value In People (VIP) award and now holds a British Heart Foundation (BHF) Intermediate Basic Science Fellowship (FS/08/26225).
